# Supplementary material for: Solution-processable, soft, self-adhesive, and conductive polymer composites for soft electronics
Source: Nat Commun. 2022 Jan 18;13:358. doi: 10.1038/s41467-022-28027-y (PMC8766561; doi:10.1038/s41467-022-28027-y)
Supplement: Supplementary file 1 — Supplementary Information [file 41467_2022_28027_MOESM1_ESM.pdf]

Supplementary Information

Solution-processable, soft, self-adhesive, and conductive polymer composites for soft electronics

Peng Tan[1,2], Haifei Wang[1,2], Furui Xiao[1], Xi Lu[1], Wenhui Shang[1], Xiaobo Deng[1], Huafeng Song[1], Ziyao Xu[1], Junfeng Cao[1], Tiansheng Gan[1], Ben Wang[1], and Xuechang Zhou[1]*

[1]College of Chemistry and Environmental Engineering, Shenzhen University, Shenzhen, P. R. China.

[2]These authors contributed equally: P. Tan and H. F. Wang

* Corresponding authors: X. C. Zhou (xczhou@szu.edu.cn)

Supplementary Table 1 | The details of various SCAPs with different PEDOT:PSS mass ratios

Mass ratio	SMS solution			Polymer solution		
	β-CD/g	Critic acid/g	H_2O/g	10% PVA/g	50%GA/μL	PEDOT:PSS/g
0.9%	0.59	1	5	1.2	2	1.25
1.8%	0.59	1	5	1.2	2	2.5
3.6%	0.59	1	5	1.2	2	5
7.1%	0.295	0.5	2.5	0.6	1	5
13.2%	0.295	0.5	2.5	0.6	1	10
23.3%	0.295	0.5	2.5	0.6	1	20
36.3%	0.295	0.5	2.5	0.6	1	37.5

Supplementary Figures

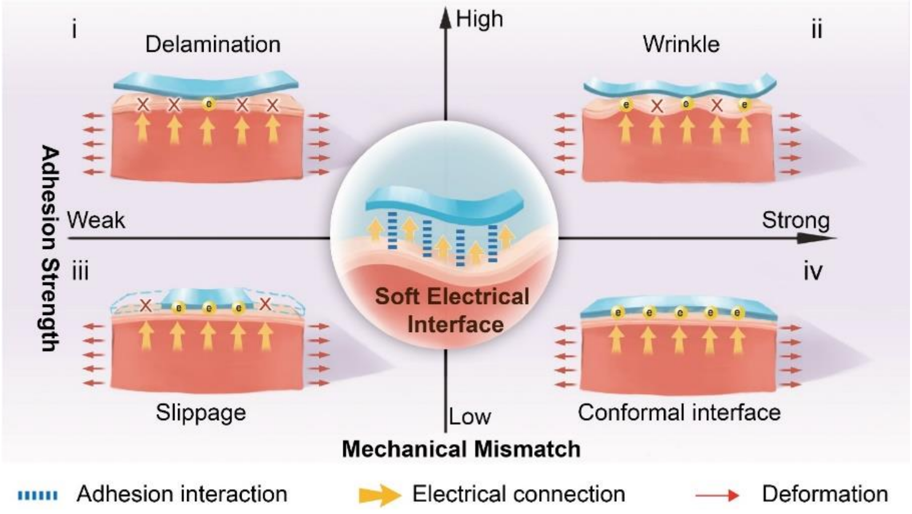

Supplementary Figure 1 | Scheme illustration of soft electrical interfaces for bridging structures during deformation with different mechanical mismatch and adhesion strength.
i, Delamination (weak adhesion and high mechanical mismatch). **ii**, Wrinkle (strong adhesion and high mechanical mismatch). **iii**, Slippage (weak adhesion and low mechanical mismatch). **iv**, Conformal interface withstanding various deformation (strong adhesion and low mechanical mismatch).

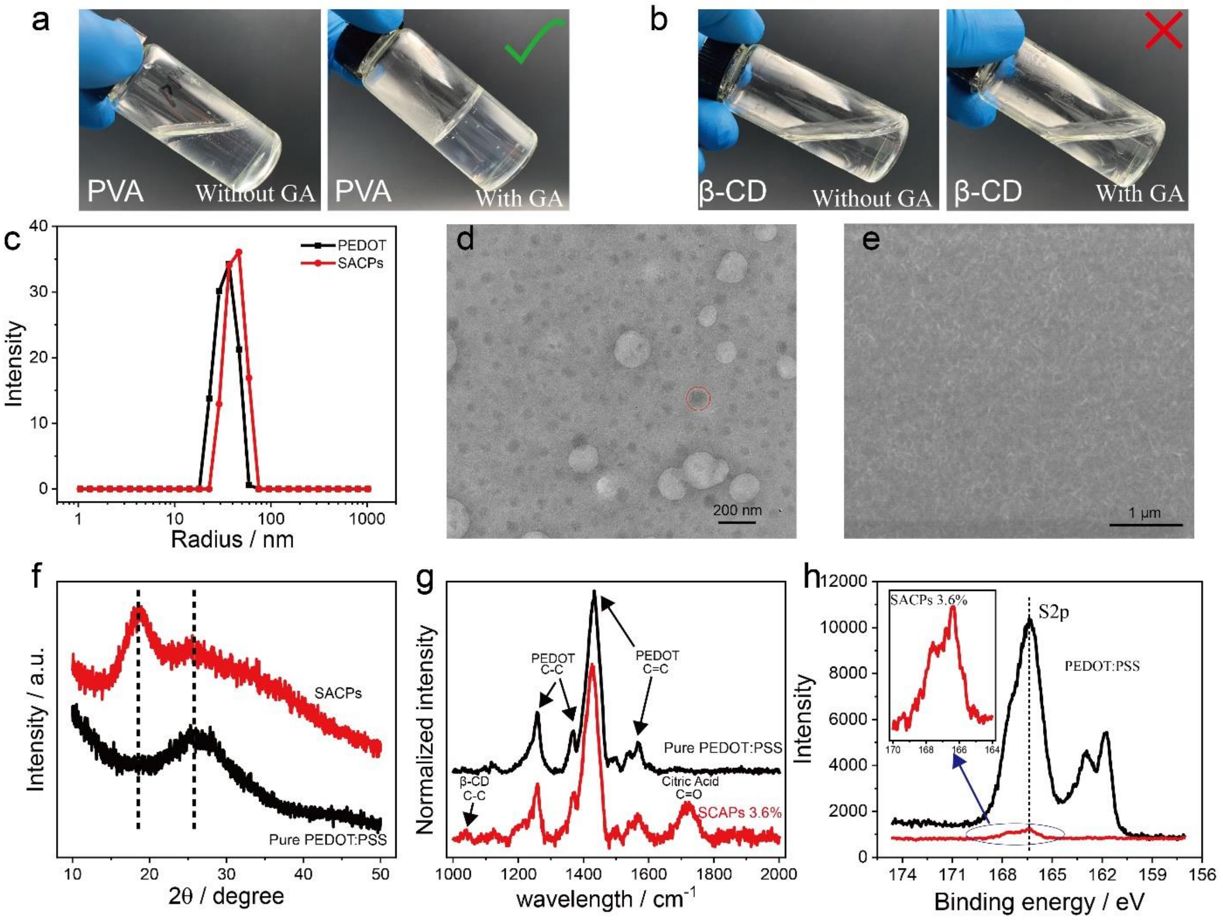

Supplementary Figure 2 | Representative chemical and microstructural characteristics of SACP composites: The reaction selectivity of -OH groups in PVA (**a**) and β-CD (**b**) for the formation of the chemically cross-linked polymer gel. **(c)** size distribution of PEDOT in pure PEDOT:PSS aqueous and SACPs solution from the tests of dynamic light scattering; TEM **(d)** and SEM (**e**) images of SCAPs; **(f)** XRD analysis of pure PEDOT:PSS film and SACPs film; **(g)** Raman spectrum of pure PEDOT:PSS and SACPs film with 3.6% mass ratio of PEDOT:PSS; **(h)** The binding energy of S_{2p} from XPS spectrum of pure PEDOT:PSS (black line) and SACPs film (red line).

According to Supplementary Figure 2a and 2b, PVA polymer solution was gelated after the addition of GA. While β-CD can't be crosslinked by GA. This is because cyclodextrin is a typical class of oligosaccharides with chair conformation, while PVA is a linear chain structure. GA reacts simultaneously with two -OH groups to form O-C-O structure. Therefore, the chemically crosslinked polymer networks were formed from PVA chains.

By the introduction of SMS, the interactions between SMS and PEDOT chain (Electrostatic action and hydrogen bonding action) help to reduce PEDOT interchain interactions and to reduce aggregation. At the same concentration of PEDOT:PSS, the good dispersion of PEDOT will improve the uniformity of film and increase conductivity. However, the conductivity of SACPs still follows the percolation theory. To present a further morphological and structural characterization of the SACPs, we carried out the dynamic light scattering (DLS), Raman, XRD, and XPS tests. As shown in Supplementary Figure 2c, the particle size distribution of PEDOT:PSS solution was slightly changed after the addition of SMS, indicating that SMS had no negative effects on the dispersion stability of PEDOT. This result

was also proved from TEM and SEM images (Supplementary Figure 2d and e). The domain size of PEDOT:PSS was about 40 nm. According to the XRD test, the diffraction peak was left shift from 25 ° to 18° as the addition of SMS, indicating an increase of the corresponding space of PEDOT (Supplementary Figure 2d and e). The aggregation of PEDOT was significantly reduced in SACPs because of the good dispersion stability of PEDOT after the addition of SMS. The elements and interactions were also confirmed by the Raman spectrum (Supplementary Figure 2g) and XPS analysis (S_{2P}) (Supplementary Figure 2h).

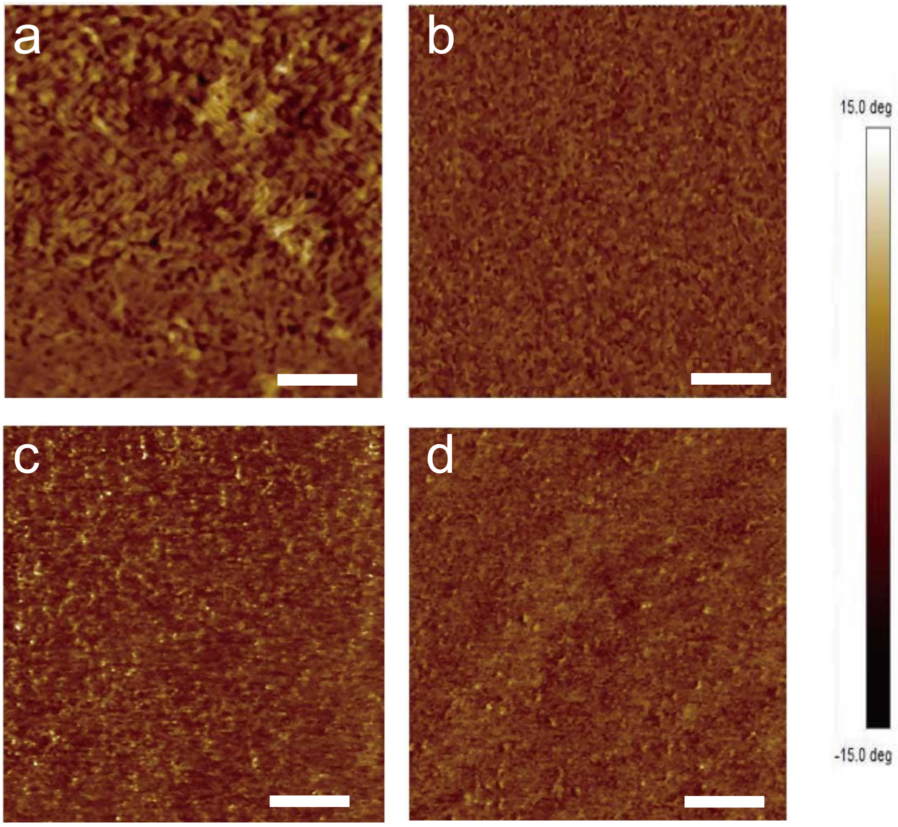

Supplementary Figure 3 | Relationships of microstructures of PEDOT:PSS and supramolecular solvent. AFM phase images of pure PEDOT:PSS (**a**) and supramolecular solvents doped PEDOT:PSS with a concentration of 13.2% (**b**), 3.6% (**c**) and 0.94% (**d**). With the increase of supramolecular solvent in content, the AFM phase images change obviously. The higher percentage of supramolecular solvents doped in PEDOT:PSS present continuous phase structures indicating that supramolecular solvents can accommodate the aggregation of PEDOT polymer chains. The weak interactions (hydrogen bonding interactions and electrostatic interactions) between supramolecular solvents and PEDOT:PSS chains play an important role in improving their aggregation structures.

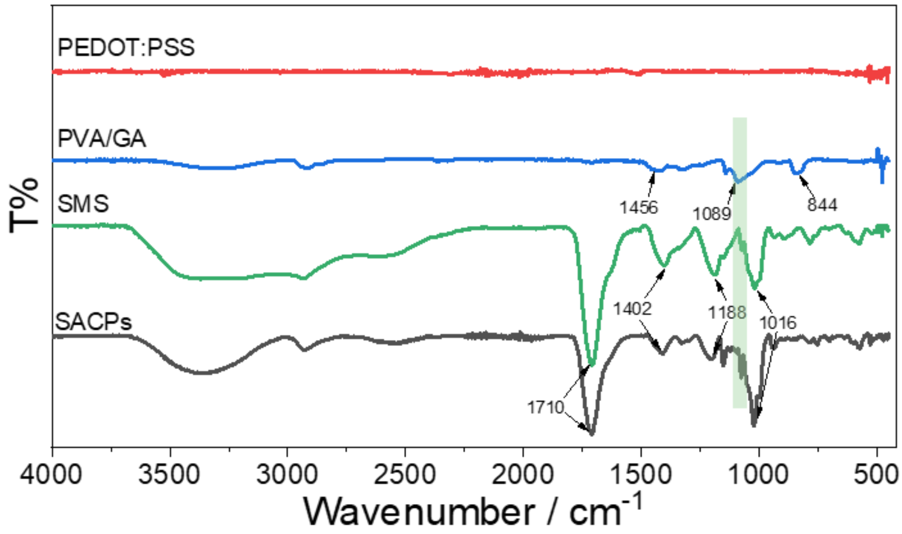

Supplementary Figure 4 | The FT-IR spectra of PEDOT:PSS, chemically crosslinked PVA with glutaraldehyde (GA) (PVA/GA), supramolecular solvents (SMS) and SACPs. For the PVA/GA, a broad band at 1456 cm^{-1} belongs to the stretching vibration of -CH_2- of PVA, and a weak peak at 844 cm^{-1} is attributed to the bending vibration of -CH_2- of PVA. Notably, we can find a specific band at 1089 cm^{-1}, which belongs to the stretching vibration of -C-O- of PVA. For SMS, the peak at 1710 cm^{-1} belongs to the -COOH groups of citric acid, and the bands at 1016, 1188 and 1402 cm^{-1} are assigned to the stretching vibration of -C-OH groups. All the peaks in FT-IR spectra of SMS can be found in that of SACPs, indicating the presence of SMS in SACPs. Importantly, we can find a weak band at 1089 cm^{-1} specific (light green area) to PVA/GA in the SACPs, demonstrating the formation of PVA polymer networks in PEDOT:PSS.

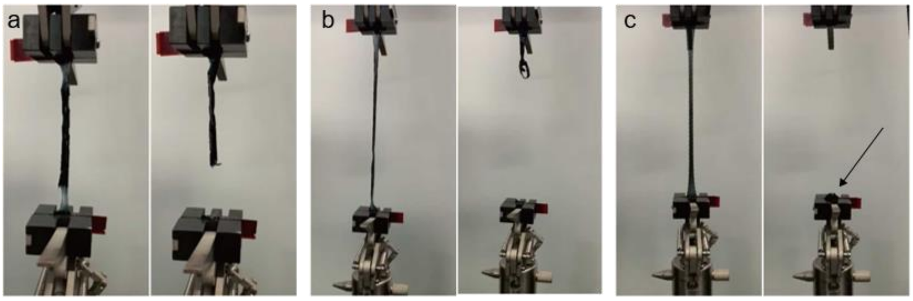

Supplementary Figure 5 | Synergistic effects of crosslinked PVA elastic networks and supramolecular on the stretchability of PEDOT:PSS composites. Optical images of stretched and fractured samples. Supramolecular solvent doped PDEOT:PSS without PVA **(a)**, with uncross linked PVA **(b)** and crosslinked PVA **(c)**. PEDOT:PSS composites doped by supramolecular solvents exhibit obviously plastic deformation, and the fractured sample was is stretched irreversibly **(a)**. With the addition of PVA networks, the fractured sample was partially recovered **(b)**. While, the introduction of crosslinked PVA elastic networks, the fractured sample can fully recover **(c)**.

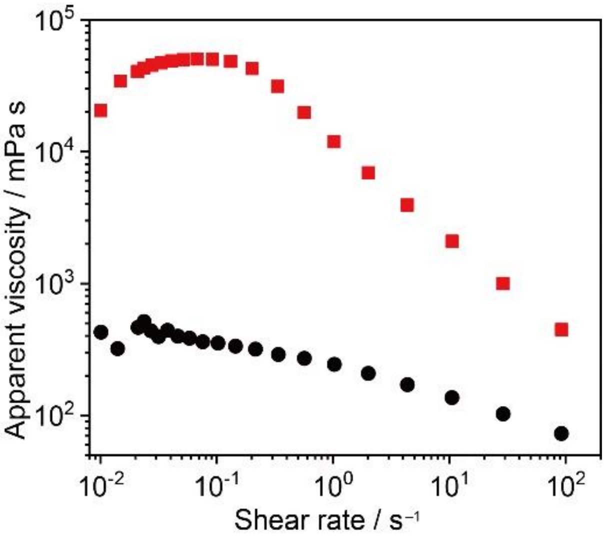

Supplementary Figure 6 | Apparent viscosity analysis of supramolecular solvent doped PEDOT:PSS. Viscosity of PEDOT:PSS mixture solution (black) has a marked increase after glutaraldehyde crosslinking reaction for 2 hours (red) under shear rate from 0.01 to 100 s^{-1}.

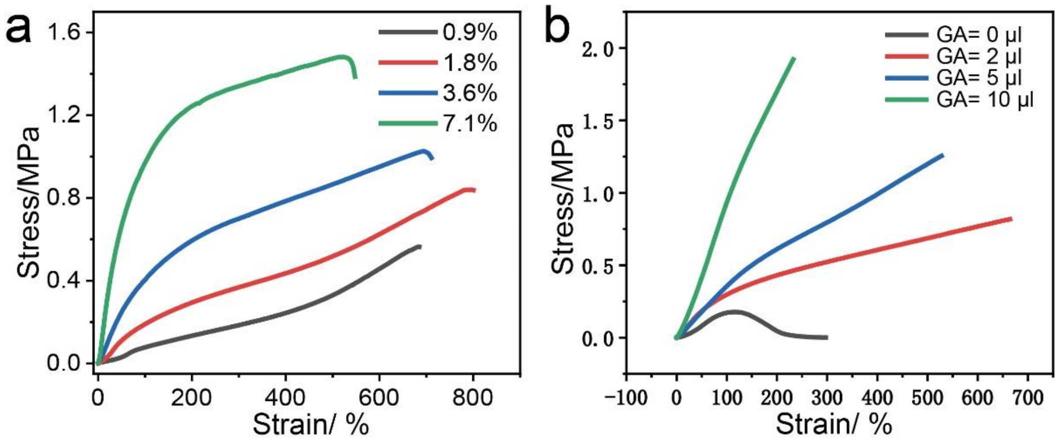

Supplementary Figure 7 | (a) Stress-strain curves of SACP films with different PEDOT:PSS concentrations. **(b)** Stress-strain curves of samples with different content of glutaraldehyde (GA). The GA is a crosslinker, and can react with OH groups in PVA networks. The yield stress is lower than 0.2 MPa for samples with uncrosslinked PVA networks, indicating plastic deformation under stretching (black line). The stretchability of the samples was improved significantly after the formation of a crosslinked PVA elastic network.

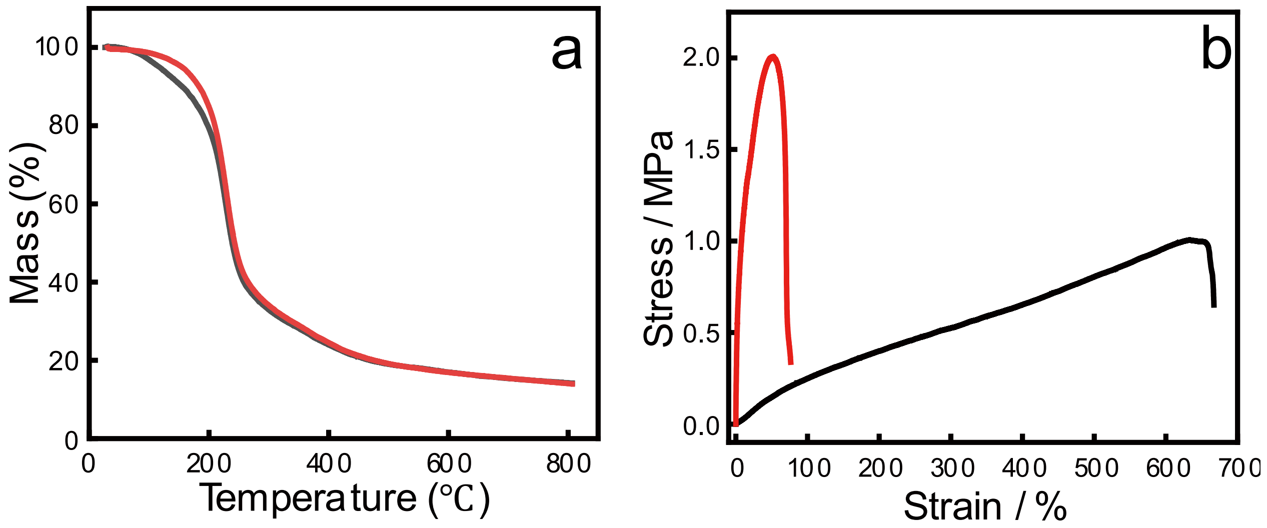

Supplementary Figure 8 | The effects of heating time on mechanical property. (a) TG curves of the SACP composite. (b) Stress-strain curves of sample under heating for 24 hours (black) and 72 hours (red). Hydrogen bonds are a class of water-sensitive weak interactions. The density of hydrogen bonds is controlled by the content of water molecules, thus affecting the strength of supramolecular interactions. Therefore, the intensity of the supramolecular interactions can be adjusted by water molecules. With increasing drying time, the water content decrease, the hydrogen bond interactions between citric acid and cyclodextrin have extremely high strength, the young's modulus of corresponding SMS doped PEDOT:PSS composites increased.

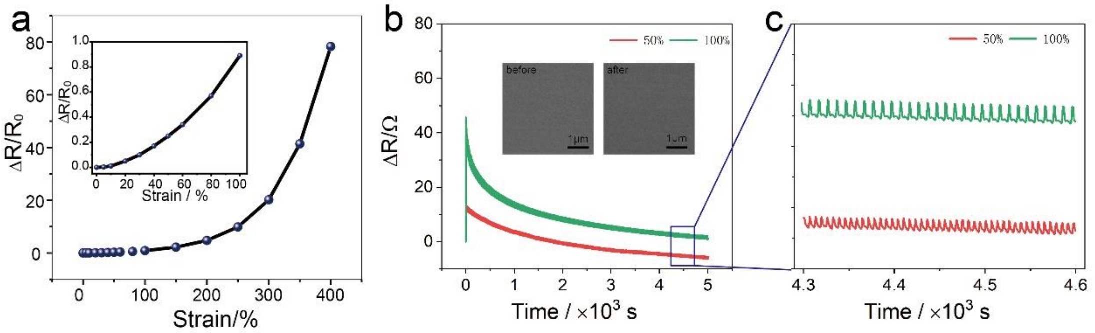

Supplementary Figure 9 | (a) The relative resistance variation ($\Delta R/R_0$) of SACP film under tensile strain. insert: Magnification of resistance-strain curve at the strain range of 0-100%. **(b) and (c)** the resistance changes of SACP film under repeated stretching-releasing cycles. Insert: SEM images of surface before and after test.

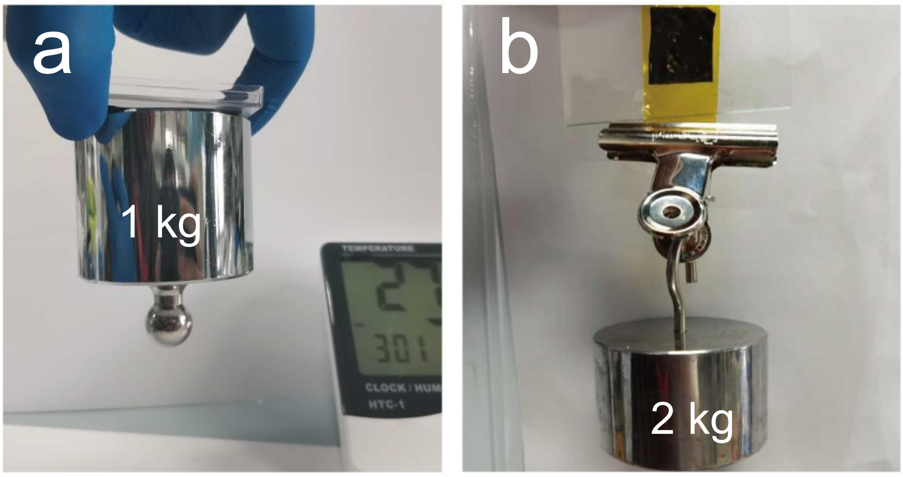

Supplementary Figure 10 | Demonstration of adhesion performance. **(a)** A 1 kg of weight was adhered to a PET substrate by the SACP film; **(b)** A 2 kg of weight was lifted by an SACP film adhered to a glass substrate.

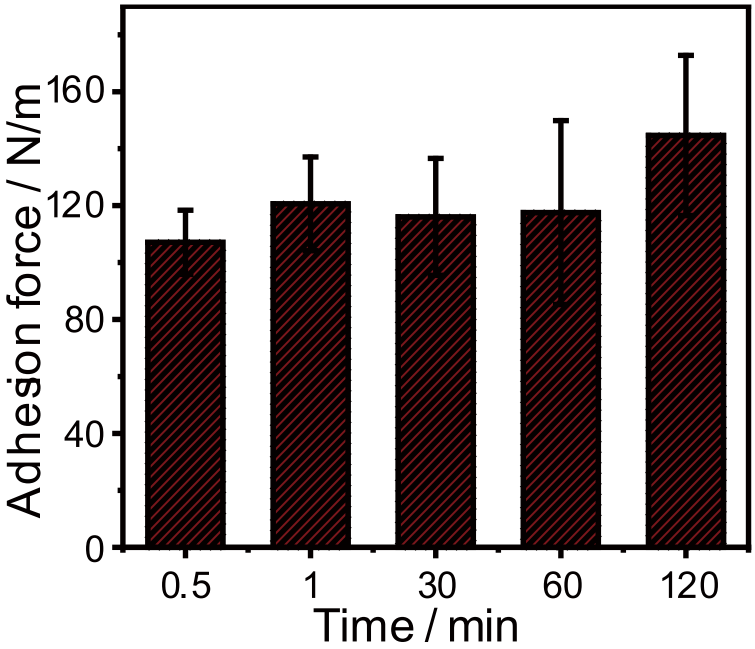

Supplementary Figure 11 | The relationship between the bonding strength and the pressing time. Stable adhesion can be achieved in a very short pressing time (< 0.5 min).

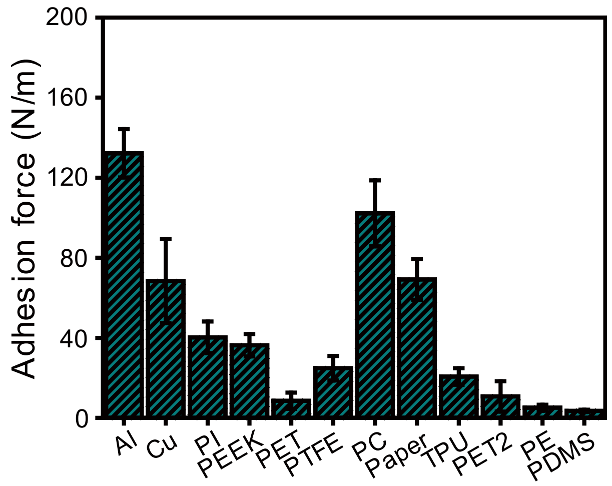

Supplementary Figure 12 | Summary of adhesion strength of commercial tapes on different substrates.

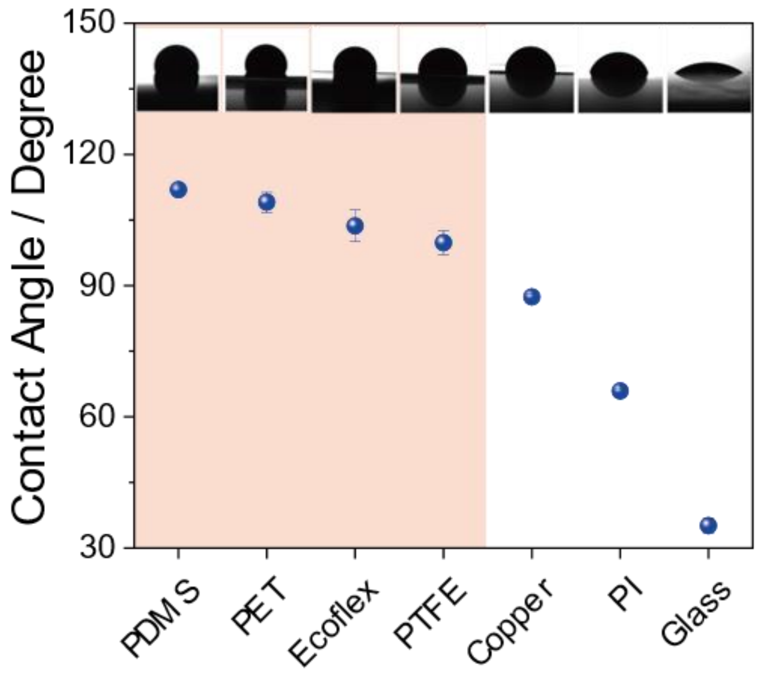

Supplementary Figure 13 | Contact angle of PEDOT: PSS mixture solution on different substrates.

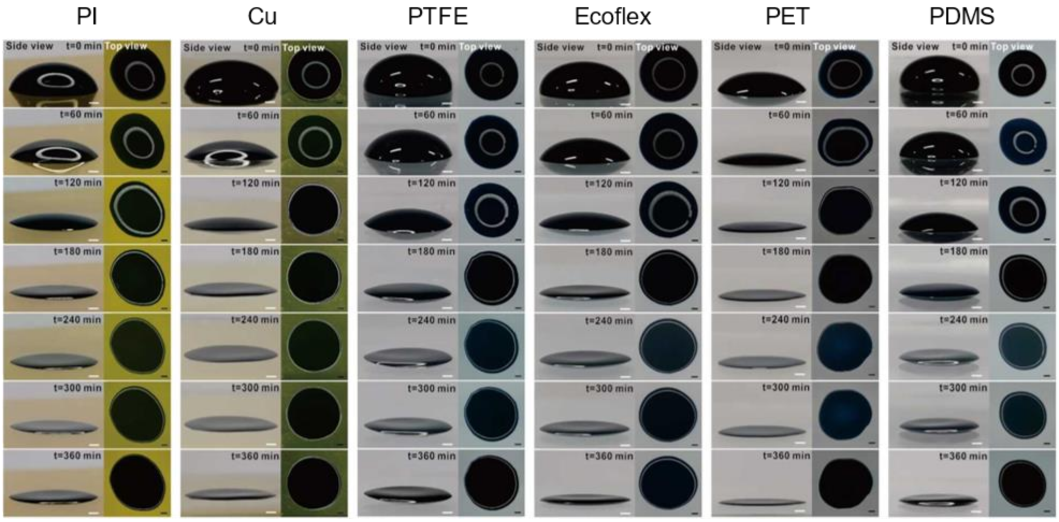

Supplementary Figure 14 | Images of drying process of SCAP film by drying PEDOT:PSS mixtures on various substrates, scale bar: 2mm.

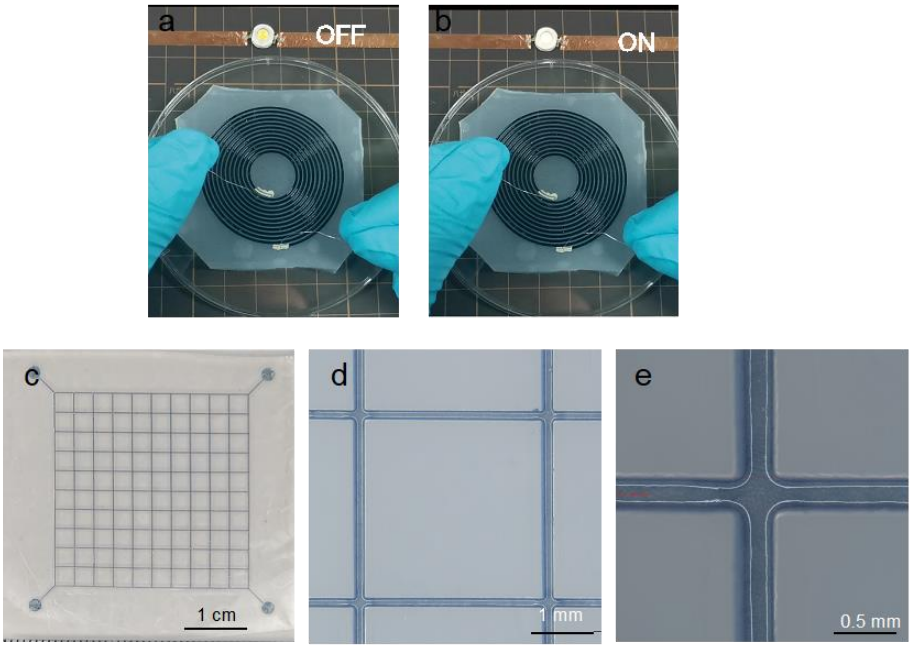

Supplementary Figure 15 | Images of conductive patterns by microfluid molding method. **a** and **b**, an LED lamp was connected in a circuit with conductive helical line. **c-e**, conductive mesh prepared by microfluid molding method.

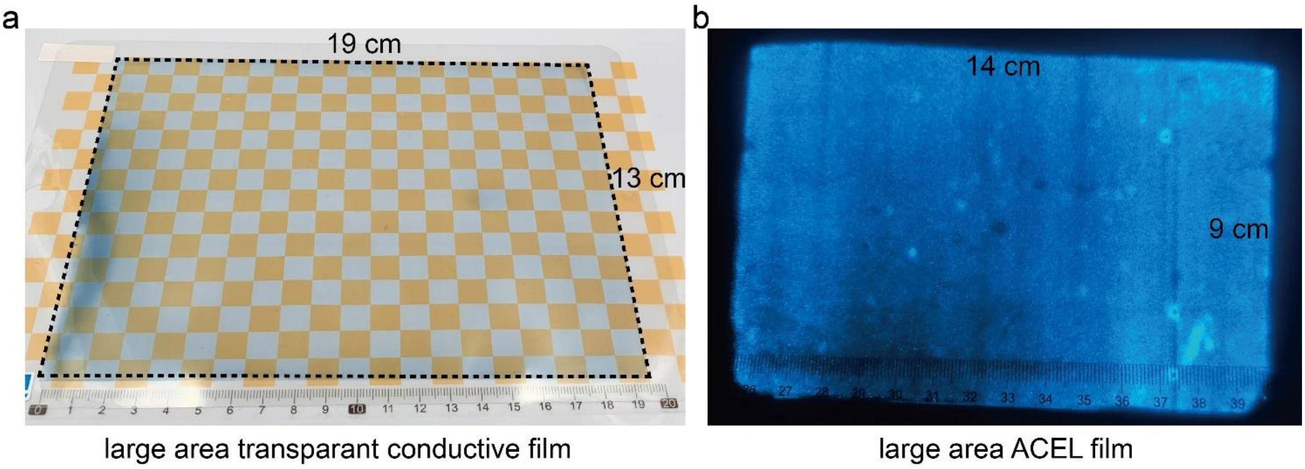

Supplementary Figure 16 | Image of a large area of self-adhesion film on PET film **(a)** coated by Meyer rods coating and the corresponding ACEL film **(b)**. A large-area SACP film (19 cm × 13 cm) was prepared by Meyer rods coating. And this large-area SACP film can be used as transparent electrode to assemble large-area ACEL film ((14 cm × 9 cm).

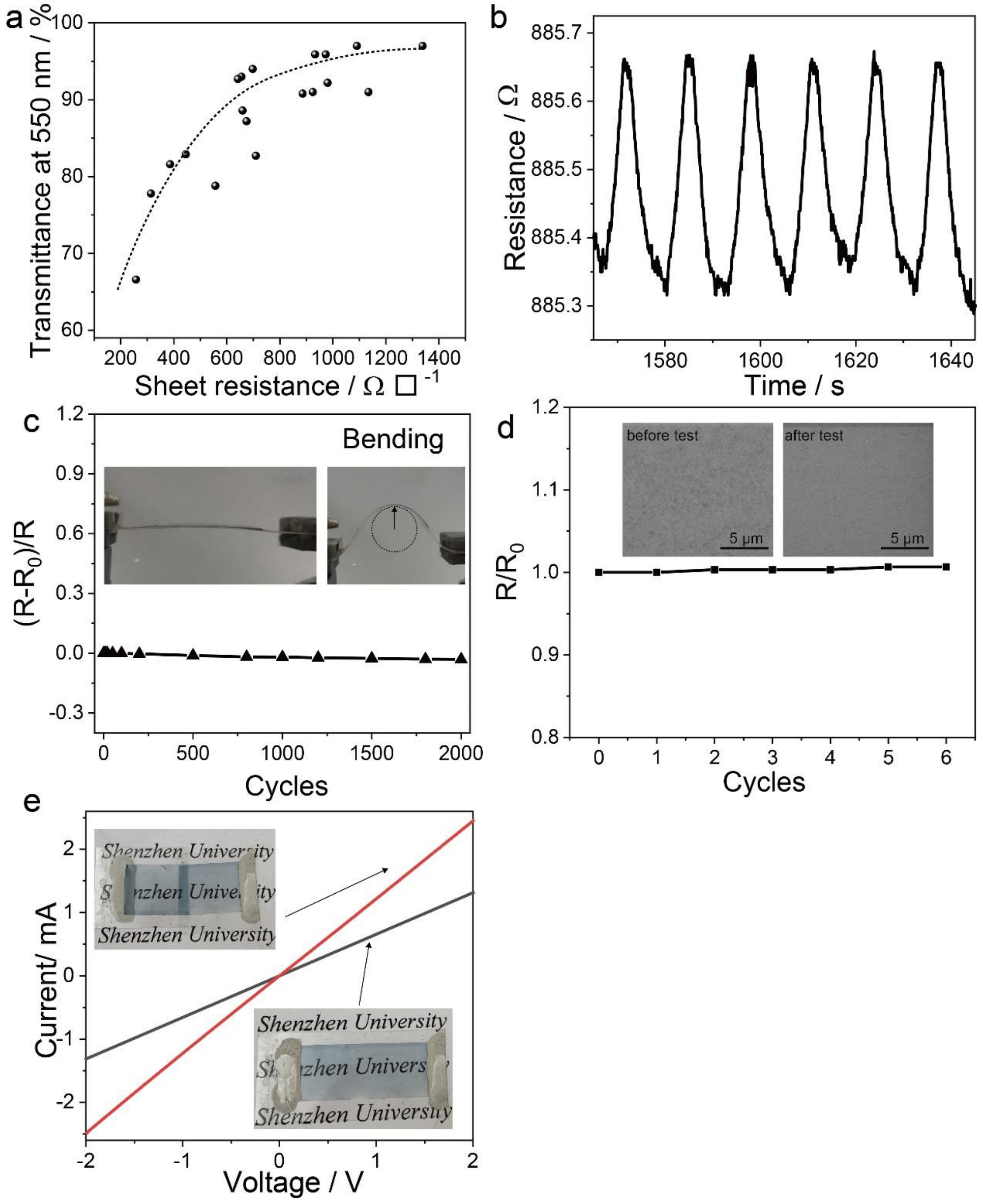

Supplementary Figure 17 | **a**, relationship of transmittance versus sheet resistance of the film. **b**, resistance changes under repeated bending tests. **c**, stability of resistance changes under cyclic bending tests with bending radius at 2 mm. **d**, the electrical resistance changes of SACP transparent conductive film under 6 cycles repeating peeling tests. Insert: SEM images of surface before and after peeling test. **e**, current-potential curves of the self-adhesion conductive film.

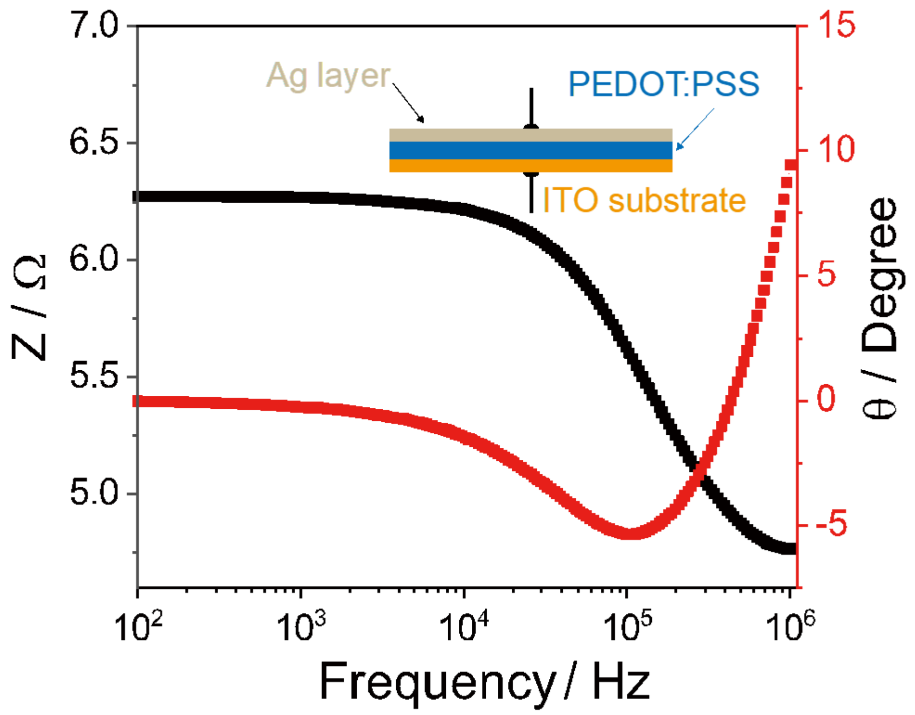

Supplementary Figure 18 | Electrical impedance spectroscopy measurements of supramolecular solvent doped PEDOT: PSS films.

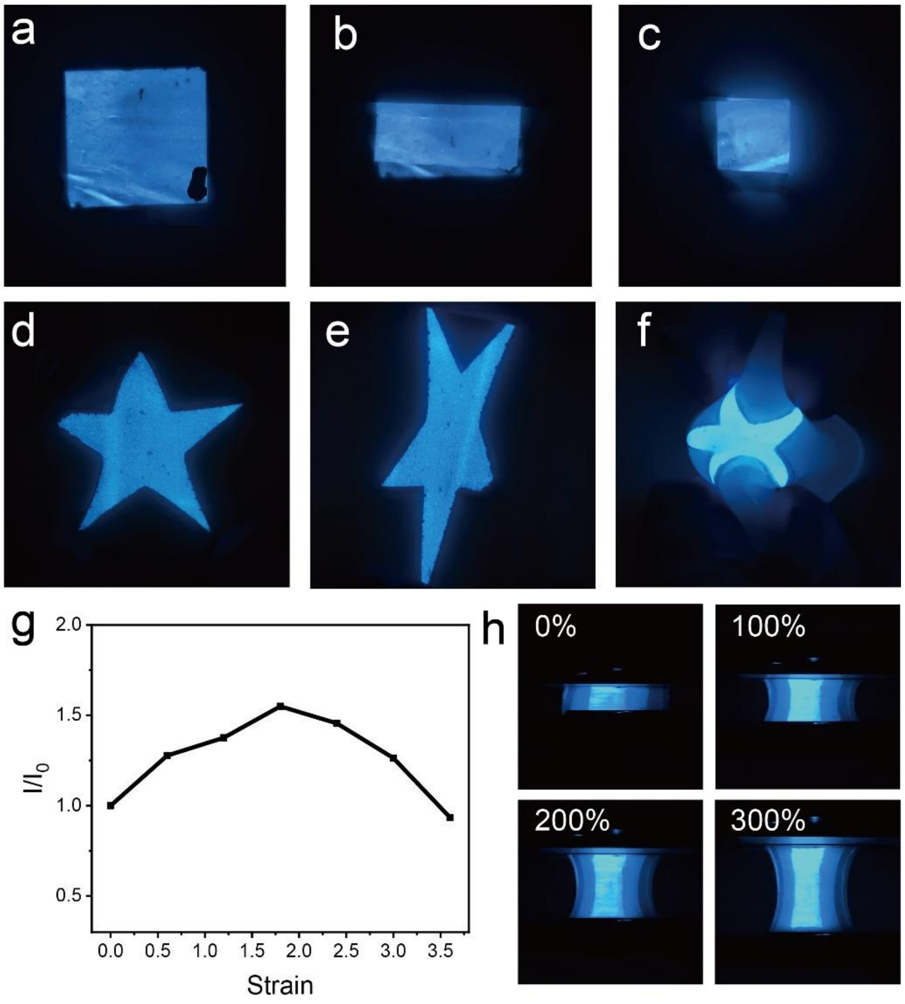

Supplementary Figure 19 | Demonstration of ACEL film under deformations. **a-c,** images of folded ACEL film in half. **d-f,** images of ACEL film under various stretching states; **(g)** The relationship of emission intensity versus strain of ACEL film; **(h)** images of ACEL film under strain of 0%, 100%, 200% and 300%.

Supplementary Note1 | Arduino code for the integrated system

```
int ledPin = 10;
int ledPin1 = 8;
int ledPin2 = 7;
int ledPin3 = 6;
int a;
void setup() {
  // put your setup code here, to run once:
 Serial.begin(115200);
 pinMode(ledPin, OUTPUT);
 pinMode(ledPin1, OUTPUT);
  pinMode(ledPin2, OUTPUT);
```

```
    pinMode(ledPin3, OUTPUT);
}

void loop() {
  // put your main code here, to run repeatedly:
  a = analogRead(A0);
  delay(200);
if (a > 350) // a threshold value to define the switches of ACEL array
{ digitalWrite(ledPin, HIGH);
digitalWrite(ledPin1, HIGH);
digitalWrite(ledPin2, HIGH);
digitalWrite(ledPin3, HIGH);// ACEL array controlling
}
else if (a >300)
{ digitalWrite(ledPin1, HIGH);
digitalWrite(ledPin2, HIGH);
digitalWrite(ledPin3, HIGH);
digitalWrite(ledPin,LOW); // ACEL array controlling
}
else if (a >270)
{ digitalWrite(ledPin2, HIGH);
digitalWrite(ledPin3, HIGH);
digitalWrite(ledPin, LOW);
digitalWrite(ledPin1,LOW);// ACEL array controlling
}
else if(a >230)
{ digitalWrite(ledPin3, HIGH);
digitalWrite(ledPin, LOW);
digitalWrite(ledPin1, LOW);
digitalWrite(ledPin2,LOW);}
else {
  digitalWrite(ledPin, LOW);
    digitalWrite(ledPin1, LOW);
      digitalWrite(ledPin2, LOW);
        digitalWrite(ledPin3, LOW);
}
 // ACEL array controlling
  }
```
