# Supplementary material for: Solution-processable, soft, self-adhesive, and conductive polymer composites for soft electronics
Source: Nat Commun. 2022 Jan 18;13:358. doi: 10.1038/s41467-022-28027-y (PMC8766561; doi:10.1038/s41467-022-28027-y)
Supplement: Supplementary file 3 — Description of Additional Supplementary Files [file 41467_2022_28027_MOESM3_ESM.pdf]

Description of Additional Supplementary Files

Supplementary Movie 1: Relationships of mechanical elasticity and PVA networks. SMS has obvious doping effects on PEDOT: PSS, resulting in a decline in mechanical rigidity and ductility. Without the help of elastic polymer (PVA) networks, SMS doped PEDOT: PSS presented typically plastic deformation. While the elasticity of SACPs was greatly improved and residual strain significantly decrease after the introduction of crosslinked PVA networks.

Supplementary Movie 2: Differences of interface adhesion of pure PEDOT: PSS and SACP films. Pure PEDOT: PSS film showed poor interface adhesion, and can be easily peeled off from the substrate. The present SACP film exhibited high adhesion performance on PI and elastic Ecoflex substrates and had stable adhesion on substrates even suffered extreme deformation.

Supplementary Movie 3: Interface adhesion of SACP films on various substrates. The SACP film exhibited good elastic resilience. A 500 g weight was pulled forward by lightly putting an SACP film on a polymer substrate. Pre-stretched SACP film can bend TPU, PTFE, PI, and PEEK ribbons into rings by lightly adhering it onto polymer substrates, indicating that SACP showed very well adhesion ability.

Supplementary Movie 4: Electronic performance of patterned SACP films. By depositing SACP into microchannel through microfluid molding, the resultant helical line was good conductive. In addition, a transfer-printed SACP circuit exhibited stable adhesion which can withstand strong dynamic deformation.

Supplementary Movie 5: Demonstration of flexibility and stability of ACEL film. The ACEL film can be kneaded and folded like paper, be rolled into a coil on a glass rod, and flap in wind, exhibiting good flexibility. Besides, it can also work well in water.

Supplementary Movie 6: Demonstration of detection of biological EMG signals. As an adhesive electrode, SACP was attached to the skin of a tester. EMG signals were precisely detected from body motions, including finger motion, gripping, lifting dumbbell, push-up, squatting, and jumping.

Supplementary Movie 7: Demonstration of an integrated system of EMG visualization detection
